# Improved diagnostic test for *Clostridium difficile:* clinical and infection control implications

**DOI:** 10.1186/1753-6561-5-S6-P182

**Published:** 2011-06-29

**Authors:** J Wilson, E Brannigan, A Jepson, C Johnstone, S Hassall, A Holmes

**Affiliations:** 1 Infection prevention & control, Imperial College Healthcare NHS Trust, London, UK

## Introduction / objectives

*Clostridium difficile* reporting is mandatory in England, numbers have increased since the mid-1990s with over 25 000 reported per year. At Imperial College Healthcare it was observed that isolation of *C. difficile* from stool did not always reflect clinical disease; particularly when testing was changedto detection of glutamate dehydrogenase (GDH) and the *tcdB* gene by PCR. Testing for *C. difficile* in liquid stools was not restricted. All positive patients were therefore reviewed to determine if they had *C. difficile* associated disease (CDAD).

## Methods

A patient was considered to have CDAD if they had at least 3 episodes of diarrhoea in 24 hours, the stool was positive for C. difficile by PCR, diarrhoea was not attributable to another cause and symptoms consistent with pseudomembranous colitis. A standardised clinical review was undertaken to determine if new cases of *C. difficile* met the case definition. Data were captured prospectively on an electronic surveillance system (ICNet).

## Results

250 patients admitted between June 2010 and January 2011 were test positive; of these, 166 (66%) were classified as hospital-acquired. Clinical review found 67 (40%) did not have CDAD: 29 (27%) did not have diarrhoea; in 80 (73%), diarrhoea was attributable to another cause, most commonly underlying bowel disease (38 cases). Severe disease occurred in 16%; there were 7 deaths associated with CDAD.

## Conclusion

This analysis indicates that improved laboratory tests for toxin-producing *C. difficile* do not correlate well with CDAD. Whilst this may be an advantage in terms of prompt initiation of infection control measures, reporting based on positive tests does not represent the true burden of disease and the laboratory method used diagnosis may have a significant impact on rates.

## Disclosure of interest

None declared.

